# Differential Effects of Estradiol on Reproductive Function in Camelids

**DOI:** 10.3389/fvets.2021.646700

**Published:** 2021-02-18

**Authors:** Rodrigo A. Carrasco, Marcelo H. Ratto, Gregg P. Adams

**Affiliations:** ^1^Department of Veterinary Biomedical Sciences, Western College of Veterinary Medicine, University of Saskatchewan, Saskatoon, SK, Canada; ^2^Department of Animal Science, Universidad Austral de Chile, Valdivia, Chile

**Keywords:** llama (*Lama glama*), estradiol, nerve growth factor, ovulation, LH

## Introduction

Based on the mechanism of ovulation, mammals may be classified as spontaneous (i.e., pig, cattle, sheep, goats) or induced ovulators (i.e., rabbit, ferret, cat, koala, llama, camel, alpaca). High or increasing concentrations of estradiol exert a positive feedback on the hypothalamus triggering an LH surge and ovulation in spontaneous ovulators, while ovulation is triggered by mating in induced ovulators. South American camelids are classified as induced ovulators but the ovulatory response is triggered by semen (1); copulation itself plays a minor role (2). Originally dubbed ovulation-inducingi factor, the factor responsible for eliciting ovulation in camelids is the protein nerve growth factor [NGF; (3–5)]. A highly conserved molecule, NGF is concentrated in camelid semen (and semen of many species), and systemic absorption from intrauterine seminal deposition has been implicated as the inciting cause of the preovulatory surge in circulating concentrations of LH after mating or parenteral administration of nerve growth factor (1, 6). Although the mechanistic pathway of NGF-induced ovulation is unknown, it is thought to be mediated at the hypothalamus instead of the pituitary gland (7). Nerve growth factor may act by binding to neurons that possess one or both NGF receptors (TrkA and P75) in the hypothalamus of llamas or by interacting with third-ventricular tanycytes, a group of cells derived from modified ependyma that possess the P75 receptor (8). A recent study using nasal placodes from mouse embryo (a source of GnRH neurons) showed that the activation of P75 signaling pathways triggered an increase in depolarization events in GnRH neurons (9), suggesting that NGF in semen may be acting at the median eminence to trigger ovulation during mating.

In a recent report (10), the effects of estradiol-17β administration on ovulation and luteal development in llamas were examined. Administration of increasing concentrations of estradiol-17β (0.6, 1, and 1.6 mg/llama) increased the ovulatory rate in an incremental manner (0/4, 1/4, and 6/6, respectively). Although LH concentrations were not reported, the ovulatory response, corpus luteum development, and plasma progesterone profile were consistent with the idea that estradiol triggers ovulation in this species. In an earlier study (11), results showed that ovariectomized llamas had an impaired response to NGF-induced LH surge, which was partially recovered by pre-treatment with estradiol. Thus, it was suggested that estradiol promotes the fullness of the pre-ovulatory LH surge induced by NGF. The report from Bianchi et al. (10), however, is the first to directly test the effects of estradiol on ovulation in llamas.

## Discussion

**Figure 1:**
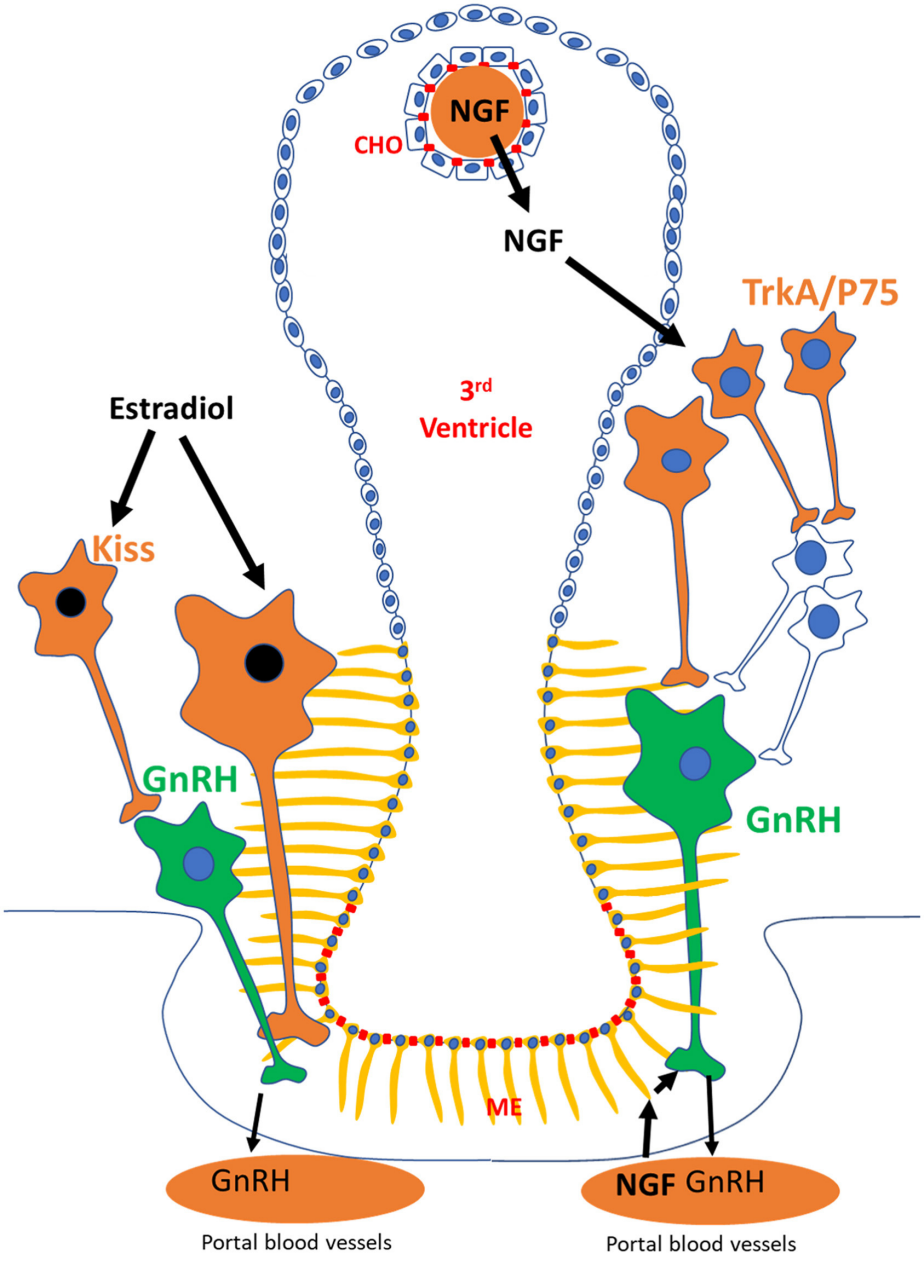
Proposed mechanistic pathways followed by estradiol and NGF in the hypothalamus of llamas to trigger ovulation. Estradiol, through neuronal populations (i.e., kisspeptin; kiss) expressing its nuclear receptor (black), may be influencing GnRH neurons (green) to trigger an LH surge. In contrast, the effects of NGF on GnRH neurons may be triggered by activation of NGF receptors (TrkA and P75) in hypothalamic neurons that act as afferents to GnRH neurons. Alternatively, blood borne NGF may be activating P75 receptor in tanycytes (yellow), which are in close association to GnRH nerve terminals at the portal vessels in the median eminence (ME). CHO, choroid plexus.

The results of Bianchi et al. (10) are consistent with previous studies in other induced ovulators. In rabbits, administration of a combination of estrogen and progesterone daily for 2 days, but not estrogen or progesterone independently, led to ovulation in 40% of synchronized does (12). In California voles, administration of estradiol-17β or estradiol benzoate caused ovulation in up to 28% of treated females (13). In these studies, however, assessment of the ovulatory response was based on single examinations (laparotomy or post-mortem), while Bianchi et al. (10) used a non-invasive approach (ultrasonography and blood sampling) that allowed clear characterization of corpus luteum form and function. Despite the limited information available in scientific literature, results point out the possibility that camelids, and other induced ovulators, may elicit an LH surge in response to both estradiol and NGF. How NGF and estradiol induce ovulation in camelids remains unknown, but we hypothesize that the routes involved are different between the molecules based on differences in chemical composition (steroid vs. polypeptide), cellular mechanism of action (nuclear vs. transmembrane receptors), and receptor distribution (Figure 1).

The results from Bianchi et al. (10) are exciting since estradiol may be stimulating neuronal pathways to trigger a GnRH and LH surge in camelids in addition to providing support for the effects of NGF (11). Based on rabbit studies (14), the principal factor driving hypothalamic discharge of GnRH into the portal vessels in induced ovulators is the conduction of physical coital stimuli via spinal cord transmission of action potentials. That llamas ovulate in response to estradiol and seminal NGF is consistent with the notion that spontaneous and induced ovulation share complimentary mechanistic pathways for generating an LH surge. The notion of complimentary pathways is reflected in observations that the presence of males facilitates the ovulatory response in spontaneous ovulators. For instance, albino rats maintained in a constant light environment were sexually receptive and females responded to mating with an LH surge and ovulation (15). In addition, introduction of rams to ewes stimulates cyclicity and ovulation during the transition from the breeding to non-breeding seasons (16), and clitoral stimulation hastened ovulation in cattle (17). Thus, neuronal systems relevant to spontaneous ovulators may play a role in induced ovulators and vice-versa. Hypothalamic kisspeptin neurons are mediators of the estradiol-induced LH surge in ewes and other spontaneous ovulators (18), and recent studies showed that kisspeptin neurons also participate in surge-release of LH in llamas (19). In llamas, however, the absence of NGF receptor expression in kisspeptin neurons suggests that kisspeptin neurons may not be the direct target of NGF in the hypothalamus (19).

In addition to an influence on gonadotrophin secretion in induced ovulators (1, 20), both estradiol and NGF may modify luteal function. For instance, llamas that ovulated in response to NGF developed a larger corpus luteum and produced more progesterone than llamas treated with GnRH (1), and administration of NGF was associated with an increase in vascularization in the dominant follicle and the consequent corpus luteum (21). In rabbits, estradiol had a luteotrophic effect by preventing apoptosis in luteal cells (22), and, conversely, one of the luteolytic mechanisms of prostaglandin F2alpha may involve blockade of estradiol signaling in luteal cells (23). Although a luteotrophic effect of estradiol was reported in a study in which llamas were treated with high doses of estradiol [10 mg daily for 9 days; (24)], the effect of estradiol on ovarian function in llamas and its relationship with NGF remains to be investigated.

Finally, the findings by Bianchi et al. (10) may provide an explanation for spontaneous ovulation in camelids, a phenomenon that has been reported to occur at a rate of 5–15% in llamas and alpacas (25–27), and 5 and 41.7% in non-lactating and lactating camels, respectively (28). Although circulating concentrations of estradiol were not reported in Bianchi's work and the dose administered of estradiol was relatively high, we infer that a sudden increase in estradiol concentration triggers an LH surge in llamas, as in spontaneous ovulators. Thus, we hypothesize that in camelids the endogenous production of ovarian estradiol does not reach the threshold necessary to trigger an “spontaneous” ovulatory response, and instead, the female camelid relies on seminal NGF to ovulate. Further knowledge is needed regarding the source of estradiol (i.e., gonadal, adrenal, or neural), the effective circulating concentrations of estradiol (or its metabolites) to promote ovulation, and the feedback mechanisms of estradiol on LH secretion in camelids. The findings from Bianchi et al. (10) open a variety of questions regarding the ovulatory process in camelids, and the potential interactions between estradiol and NGF. The llama (and other camelids) is an excellent model to study the differential effects of estradiol (negative and positive feedback) and NGF in the generation of an LH surge.

Classification of species as spontaneous or induced ovulators may be overly strict since the latter can occasionally ovulate spontaneously and ovulation in the former can occasionally be induced or accelerated by coitus. According to Conaway (29), species may be classified as (1) spontaneous ovulators in which ovulation depends on ovarian steroid production with spontaneous occurrence of a luteal phase (pig, cow), (2) spontaneous ovulators in which ovulation depends on ovarian steroid production but a luteal phase is induced by coitus (rat, mouse), or (3) induced ovulators in which coitus induces ovulation with spontaneous occurrence of a luteal phase (rabbit, camels). It is probable that, within species, the mechanism of ovulation is modulated by environmental or social factors and species represent a continuum between the extremes of strictly induced or spontaneous ovulators (30). Silva and Bianchi's work brings that to light. Perhaps the NGF pathway of induced ovulation is an evolutionary strategy for reproductive success that phylogenetically departed from spontaneous ovulation.

## Author Contributions

All participated in the conceptualization and writing of this manuscript.

## Conflict of Interest

The authors declare that the research was conducted in the absence of any commercial or financial relationships that could be construed as a potential conflict of interest.

## References

[1] Adams GP Ratto MH Huanca W Singh J. Ovulation-inducing factor in the seminal plasma of alpacas and llamas. Biol Reprod. (2005) 73:452–7. 10.1095/biolreprod.105.040097 15888733

[2] Berland MA Ulloa-Leal C Barría M Wright H Dissen GA Silva. Seminal plasma induces ovulation in llamas in the absence of a copulatory stimulus: role of nerve growth factor as an ovulation-inducing factor. Endocrinology. (2016) 157:3224–32. 10.1210/en.2016-1310 27355492 PMC4967124

[3] Ratto MH Leduc YA Valderrama XP van Straaten KE Delbaere LT Pierson RA. The nerve of ovulation-inducing factor in semen. Proc Natl Acad Sci USA. (2012) 109:15042–7. 10.1073/pnas.1206273109 22908303 PMC3443178

[4] Fatnassi MM Cebrián-Pérez J Salhi I Pérez-Pé R Seddik M Casao A. Identification of β-nerve growth factor in dromedary camel seminal plasma and its role in induction of ovulation in females. Emir J Food Agri. (2017) 29:293–9. 10.9755/ejfa.2016-11-1585

[5] El Allali K El Bousmaki N Ainani H Simonneaux V. Effect of the camelid's seminal plasma ovulation-inducing factor/β-NGF: a kisspeptin target hypothesis. Front Vet Sci. (2017) 4:99. 10.3389/fvets.2017.00099 28713816 PMC5491598

[6] Ratto MH Huanca W Singh J Adams GP. Local versus systemic effect of ovulation-inducing factor in seminal plasma of alpacas. Reprod Biol Endocrinol. (2005) 3:29. 10.1186/1477-7827-3-29 16018817 PMC1190216

[7] Silva ME Smulders JP Guerra M Valderrama XP Letelier C Adams GP. Cetrorelix suppresses the preovulatory LH surge and ovulation induced by ovulation-inducing factor (OIF) present in llama seminal plasma. Reprod Biol Endocrinol. (2011) 30:74. 10.1186/1477-7827-9-74 21624125 PMC3123631

[8] Carrasco RA Singh J Ratto MH Adams GP. Neuroanatomical basis of the nerve growth factor ovulation-induction pathway in llamas. Biol Reprod. (2020). 10.1093/biolre/ioaa223. [Epub ahead of print]. 33331645

[9] Pinet-Charvet C Fleurot R Derouin-Tochon F de Graaf S Druart X Tsikis G. Beta-nerve growth factor stimulates spontaneous electrical activity of *in vitro* embryonic mouse GnRH neurons through a P75 mediated-mechanism. Sci Rep. (2020) 10:10654. 10.1038/s41598-020-67665-4 32606357 PMC7326925

[10] Bianchi CP Benavente MA Viviani F Gallelli MF Aba MA. Estradiol-17β injection induces ovulation in llamas. Front Vet Sci. (2020) 7:576204. 10.3389/fvets.2020.576204 33195576 PMC7593481

[11] Silva ME Recabarren MP Recabarren SE Adams GP Ratto MH. Ovarian estradiol modulates the stimulatory effect of ovulation-inducing factor (OIF) on pituitary LH secretion in llamas. Theriogenology. (2012) 77:1873–82. 10.1016/j.theriogenology.2012.01.004 22401833

[12] Sawyer CH Everett JW Markee JE. “Spontaneous” ovulation in the rabbit following combined estrogen-progesterone treatment. Proc Soc Exp Biol Med. (1950) 74:185–6. 10.3181/00379727-74-17848 15430428

[13] Milligan SR. The feedback of exogenous steroids on LH release and ovulation in the intact female vole (Microtus agrestis). J Reprod Fertil. (1978) 54:309–11. 10.1530/jrf.0.0540309 364048

[14] Kaynard AH Pau KY Hess DL Spies HG. Gonadotropin-releasing hormone and norepinephrine release from the rabbit mediobasal and anterior hypothalamus during the mating-induced luteinizing hormone surge. Endocrinology. (1990) 127:1176–85. 10.1210/endo-127-3-1176 2117523

[15] Brown-Grant K Davidson JM Greig F. Induced ovulation in albino rats exposed to constant light. J Endocrinol. (1973) 57:7–22. 10.1677/joe.0.0570007 4735633

[16] Martin GB Oldham CM Cognie Y Pearce D. The physiological responses of anovulatory ewes to the introduction of rams—a review. Livest Prod Sci. (1986) 15:219–47. 10.1016/0301-6226(86)90031-X

[17] Randel RD Short RE Christensen DS Bellows RA. Effects of various mating stimuli on the LH surge and ovulation time following synchronization of estrus in the bovine. J Anim Sci. (1973) 37:128–30. 10.2527/jas1973.371128x 4737208

[18] Smith JT Li Q Yap KS Shahab M Roseweir AK Millar RP. Kisspeptin is essential for the full preovulatory LH surge and stimulates GnRH release from the isolated ovine median eminence. Endocrinology. (2011) 152:1001–12. 10.1210/en.2010-1225 21239443

[19] Carrasco RA Leonardi CE Hutt K Singh J Adams GP. Kisspeptin induces LH release and ovulation in an induced ovulator. Biol Reprod. (2020) 103:49–59. 10.1093/biolre/ioaa051 32307518

[20] Sanchez-Rodriguez A Arias-Álvarez M Millán P Lorenzo PL García-García RM Rebollar PG. Physiological effects on rabbit sperm and reproductive response to recombinant rabbit beta nerve growth factor administered by intravaginal route in rabbit does. Theriogenology. (2020) 157:327–34. 10.1016/j.theriogenology.2020.08.003 32836052

[21] Ulloa-Leal C Bogle OA Adams GP Ratto MH. Luteotrophic effect of ovulation-inducing factor/nerve growth factor present in the seminal plasma of llamas. Theriogenology. (2014) 81:1101–7. 10.1016/j.theriogenology.2014.01.038 24582374

[22] Goodman SB Kugu K Chen SH Preutthipan S Tilly KI Tilly JL. Estradiol-mediated suppression of apoptosis in the rabbit corpus luteum with a shift in expression of Bcl-2 family members favoring cellular survival. Biol Reprod. (1998) 59:820–7. 10.1095/biolreprod59.4.820 9746731

[23] Maranesi M Zerani M Lilli L Dall'Aglio C Brecchia G Gobbetti A. Expression of luteal estrogen receptor, interleukin-1, and apoptosis-associated genes after PGF2alpha administration in rabbits at different stages of pseudopregnancy. Domest Anim Endocrinol. (2010) 39:116–30. 10.1016/j.domaniend.2010.03.001 20427144

[24] Powell SA Smith BB Timm KI Menino AR Jr. Estradiol production by preimplantation blastocysts and increased serum progesterone following estradiol treatment in llamas. Anim Reprod Sci. (2007) 102:66–75. 10.1016/j.anireprosci.2006.10.002 17116376

[25] Fernandez-Baca S Madden DH Novoa C. Effect of different mating stimuli on induction of ovulation in the alpaca. J Reprod Fertil. (1970) 22:261–7. 10.1530/jrf.0.0220261 5464117

[26] Adams GP Sumar J Ginther OJ. Form and function of the corpus luteum in llamas. Anim Reprod Sci. (1991) 24:127–38. 10.1016/0378-4320(91)90088-H

[27] Sumar JB. Reproduction in female South American domestic camelids. J Reprod Fertil Suppl. (1999) 54:169–78. 10692853

[28] Nagy P Juhasz J Wernery U. Incidence of spontaneous ovulation and development of the corpus luteum in non-mated dromedary camels (*Camelus dromedarius*). Theriogenology. (2005) 64:292–304. 10.1016/j.theriogenology.2004.11.020 15955354

[29] Conaway CH. Ecological adaptation and mammalian reproduction. Biol Reprod. (1971) 4:239–47. 10.1093/biolreprod/4.3.239 5000279

[30] Kauffman AS Rissman EF. Neuroendocrine control of mating-induced ovulation. In: Neill JD, editor. Knobil and Neill's Physiology of Reproduction. Cambridge, MA: Academic Press (2006). p. 2283–326.

